# POPX2 phosphatase regulates apoptosis through the TAK1-IKK-NF-*κ*B pathway

**DOI:** 10.1038/cddis.2017.443

**Published:** 2017-09-14

**Authors:** Ting Weng, Cheng-Gee Koh

**Affiliations:** 1 School of Biological Sciences, Nanyang Technological University, Singapore 637551, Singapore; 2 Mechanobiology Institute, Singapore 117411, Singapore

## Abstract

Chemoresistance is one of the leading causes that contributes to tumor relapse and poor patient outcome after several rounds of drug therapy. The causes of chemoresistance are multi-factorial. Ultimately, it is the balance of pro- and anti-apoptotic activities in the cells. We have previously reported links between POPX2 serine/threonine phosphatase with cell motility and invasiveness of breast cancer cells. Here, we show that POPX2 plays a role in the regulation of apoptosis. The effect of POPX2 on apoptosis centers on the inactivation of TGF-*β* activated kinase (TAK1). TAK1 is essential for several important biological functions including innate immunity, development and cell survival. We find that POPX2 interacts directly with TAK1 and is able to dephosphorylate TAK1. Cells with lower levels of POPX2 exhibit higher TAK1 activity in response to etoposide (VP-16) treatment. This subsequently leads to increased translocation of NF-*κ*B from the cytosol to the nucleus. Consequently, NF-*κ*B-mediated transcription of anti-apoptotic proteins is upregulated to promote cell survival. On the other hand, cells with higher levels of POPX2 are more vulnerable to apoptosis induced by etoposide. Our data demonstrate that POPX2 is a negative regulator of TAK1 signaling pathway and modulates apoptosis through the regulation of TAK1 activity. As inhibition of TAK1 has been proposed to reduce chemoresistance and increase sensitivity to chemotherapy in certain types of cancer, modulation of POPX2 levels may provide an additional avenue and consideration in fine-tuning therapeutic response.

Chemotherapeutics are extensively and routinely used in cancer treatment. The effectiveness of chemotherapy relies on the capability of therapeutic agents to initiate apoptosis in the tumor cells. However, some cancer cells inevitably escape apoptosis as alterations in their signaling profiles enable them to survive the drug treatment, resulting in poor prognosis of the patients. Therefore, insights into the molecular mechanism of cancer drug resistance are indispensable for the development of new and effective anti-cancer therapies.

In this study we have uncovered a regulatory link between the POPX2 (Partner of PIX 2) phosphatase and apoptosis in the context of cytotoxic chemotherapeutics. POPX2 is a serine/threonine phosphatase belonging to the PP2C family. Substrates of POPX2 identified so far are p21-activated kinase (PAK), calcium/calmodulin-dependent protein kinase II (CaMKII) and kinesin family member 3A (KIF3A).^1, 2, 3^ We have previously reported that POPX2 regulates serum response factor (SRF) mediated transcription, cell polarity, and vesicle transport along the microtubules.^3, 4, 5^ High POPX2 levels in the cell are positively correlated with cell motility and invasiveness,^6^ possibly due to modulation of glycogen synthase kinase 3 (GSK3*α/β*) and mitogen-activated protein kinases (MAPK1/3) activities.^7, 8^ However, our recent publication has associated angiogenesis as well as cancer metastasis with POPX2 levels at later stages of tumorigenesis.^9^

POPX2 phosphatase is an important regulator of a myriad of biological processes. It influences signaling pathways through its interaction with different partners. Here we report TGF-*β*-activated kinase 1 (TAK1) as a new interacting partner and a substrate of POPX2. TAK1, a kinase playing essential roles in innate and adaptive immune responses, has been found to be a powerful pro-survival molecule. TAK1, also known as mitogen-activated protein kinase kinase kinase 7 (MAP3K7), is activated by cytokines such as interleukin-1 (IL-1), tumor necrosis factor*-α* (TNF-*α*), and pathogen-associated molecular patterns including lipopolysaccharide (LPS).^10, 11, 12^ Activation of TAK1 requires complex formation with its binding proteins TAB1 (TAK1-binding protein 1) and TAB2 or TAB3.^13, 14, 15^ The binding of TAB1 to the kinase domain of TAK1 promotes autophosphorylation of TAK1, whereas association of TAB2 or TAB3 to the C-terminal region of TAK1 facilitates oligomerization of the complex and its downstream effector I*κ*B Kinase (IKK) complex.^16, 17, 18, 19, 20^ TAK1 is well-documented to activate the IKK-NF-*κ*B pathway. Sequestration of NF-*κ*B complex by I*κ*B in the cytosol switches off the transcriptional function of NF-*κ*B.^21^ However, TAK1 activation by stressors such as cytokine, ionizing radiation and exposure to genotoxin triggers phosphorylation of I*κ*B via the IKK complex, which contributes to the degradation of I*κ*B, thus releasing NF-*κ*B to translocate to the nucleus.^22, 23, 24^ TAK1 protects the cells from entering apoptosis by switching on NF-*κ*B-mediated transcription, leading to upregulation of anti-apoptotic proteins.

Our investigation into the physiological role of POPX2-TAK1 interaction suggests that POPX2 dephosphorylates and inactivates TAK1. This in turn affects the anti-apoptotic activities of the TAK1 pathway. Therefore, POPX2 is able to regulate etoposide induced apoptosis through the modulation of NF-*κ*B activities.

## Results

### POPX2 interacts with TAK1

**Figure 1:**
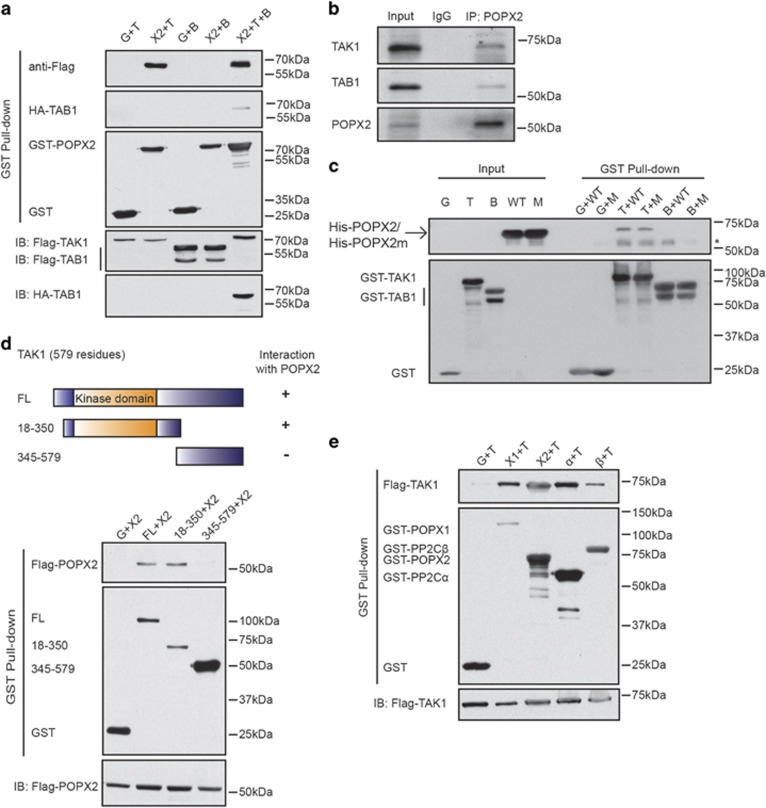
TAK1 interacts with POPX2. (**a**) GST or GST-POPX2 was co-expressed with Flag-TAK1, Flag-TAB1 or Flag-TAK1 and HA-TAB1 in COS-7 cells. Proteins pulled down from the cell lysates using glutathione sepharose beads were subjected to SDS-PAGE and Western blot analysis. G, GST; X2, POPX2; T, TAK1; B, TAB1. (**b**) POPX2 was isolated using anti-POPX2 mouse monoclonal antibody (Abcam) from U-2OS cells. Proteins co-immunoprecipitated with POPX2 were detected with indicated antibodies. Mouse random IgG was used to demonstrate absence of non-specific binding. True Blot anti-mouse and anti-rabbit secondary antibodies were used to avoid detection of IgG. (**c**) Bacterial expressed His-POPX2 or His-POPX2m was incubated with bacterial expressed GST, GST-TAK1, or GST-TAB1 bound to glutathione sepharose beads, followed by stringent wash. The input contains 10% of the total proteins subjected to GST pulldown. WT, POPX2; M, POPX2m. * indicates non-specific band. (**d**) Plasmids encoding GST, or GST tagged TAK1 (full-length and different truncated constructs) were co-transfected with Flag-POPX2 into COS-7 cells. The cell lysates were subjected to GST pulldown and proteins isolated were analyzed by Western blot. (**e**) Flag-TAK1 was co-expressed with GST and GST tagged protein phosphatases as indicated in COS-7 cells. Complexes isolated from the cell lysates by glutathione sepharose beads were subjected to western blot analysis. X1, POPX1; X2, POPX2; *α*, PP2C*α*; *β*, PP2C*β*

TAB1 (TAK1-binding protein 1), was identified in the POPX2 complex in a pulldown/mass spectrometry analysis using Flag-POPX2 as bait. As TAB1 usually associates with TAK1 and facilitates activation of this kinase,^14, 20, 25, 26^ it raised the possibility that POPX2 may also associate with TAK1 or TAK1 complex. To confirm the interactions of TAK1 or TAB1 with POPX2, proteins recovered from GST-POPX2 pulldown were assayed by Western blot analysis. Interestingly, Flag-TAK1 but not Flag-TAB1 was present in the complex obtained from GST-POPX2 pulldown (Figure 1a). TAB1 was detected in the GST pulldown only when TAK1 was expressed together with TAB1 and POPX2 (Figure 1a). Similar results were also observed when Flag-TAK1 or TAB1 immunoprecipitates were probed for the presence of GST-POPX2 (Supplementary Figure S1b). We found that TAK1, TAB1 and POPX2 were detected in the endogenous immunoprecipitates suggesting that these proteins exist as a trimeric complex (Figure 1b and Supplementary Figure S1b). Association of bacterially expressed TAK1 with bacterially expressed POPX2 or the phosphatase-dead mutant POPX2m can also be detected. However, we failed to detect direct interaction of bacterially expressed TAB1 with bacterially expressed POPX2 or POPX2m (Figure 1c). These observations suggest that POPX2 interacts directly with TAK1, the identification of TAB1 in the mass spectrometry was probably due to TAK1-TAB1 interaction. We also found that the kinase domain of TAK1 was required for its interaction with POPX2, as a truncated fragment lacking the kinase domain failed to bind to POPX2 (Figure 1d). Besides POPX2, interactions of TAK1 with POPX1, PP2C*α* and PP2C*β* were also observed (Figure 1e). Given that TAK1 is also reported to interact with PP2C*ε*,^27^ POPX2’s interaction with TAK1 may be a shared characteristic of PP2C family protein phosphatases.

### POPX2 regulates the phosphorylation status of TAK1

**Figure 2:**
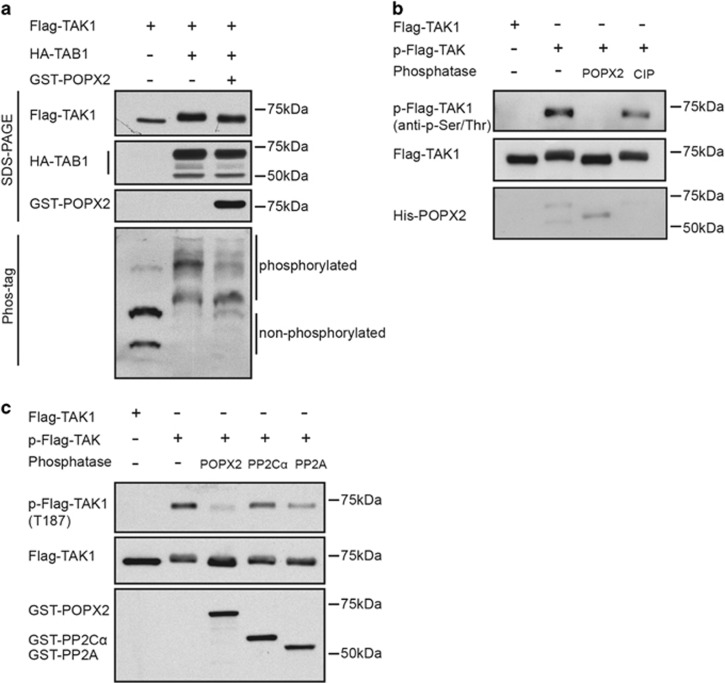
TAK1 is a substrate of POPX2. (**a**) Flag-TAK1 was expressed alone, co-expressed with HA-TAB1, or HA-TAB1 and GST-POPX2. The cell lysates harvested 24 h after transfection were analyzed using normal or Phos-tag SDS-PAGE followed by Western blot. (**b**) Flag-TAK1 or phosphorylated Flag-TAK1 (p-Flag-TAK1) was incubated with bacterial expressed His-POPX2 or calf intestinal phosphatase, CIP. The reaction mixtures were analyzed by SDS-PAGE and Western blot. p-Flag-TAK1 was detected using anti-p-Ser/Thr antibody. (**c**) p-Flag-TAK1 was incubated with GST-POPX2, GST-PP2C*α* or GST-PP2A catalytic domain harvested from COS-7 cells transfected with the respective plasmids. The reaction mixtures were analyzed by immunoblotting. Anti-p-TAK1 (T187) antibody was used to detect phosphorylation at threonine-187

Since POPX2 phosphatase interacts with TAK1, we hypothesize that POPX2 may regulate the phosphorylation status of TAK1. To test this hypothesis, a Mn^2+^-based phosphate binding tag (Phos-tag) SDS-PAGE gel was used to amplify the mobility shift of protein due to different phosphorylation status.^28^ Consistent with the previously reported findings,^14, 20^ co-expression of TAK1 with TAB1 triggered autophosphorylation of TAK1, as TAK1 bands with retarded mobility were observed in both normal and phos-tag SDS-PAGE gels (Figure 2a). The extent of the mobility shift was much reduced in the presence of POPX2 (Figure 2a), suggesting that POPX2 affects the phosphorylation status of TAK1. To determine if POPX2 directly dephosphorylate TAK1, we conducted *in vitro* phosphatase assays using bacterially expressed POPX2. The assay showed that phosphorylated TAK1 was efficiently dephosphorylated by POPX2, whereas calf-intestinal phosphatase (CIP), which was used as a control, failed to exert such significant dephosphorylation of phospho-TAK1 (Figure 2b). These observations indicate that TAK1 is a possible substrate of POPX2 phosphatase. Because phosphorylation of TAK1 at residue Thr187 is crucial for its activation,^29, 30^ we next determined if POPX2 could target TAK1 Thr187 and lead to decreased activity of TAK1. GST-tagged phosphatases were pulled down from mammalian cell lysates, we found that POPX2 was efficient in dephosphorylating TAK1 at Thr187 (Figure 2c). PP2A is used as a positive control for phosphatase activity as PP2A has been reported to dephosphorylate TAK1 and downregulate TAK1 activity.^31^ In addition, we have also confirmed that cells overexpressing POPX2 exhibited lower phospho-TAK-Thr187 induced by co-expression of TAK1 and TAB1 (Supplementary Figure S2).

### POPX2 is able to regulate VP-16-induced apoptosis

**Figure 3:**
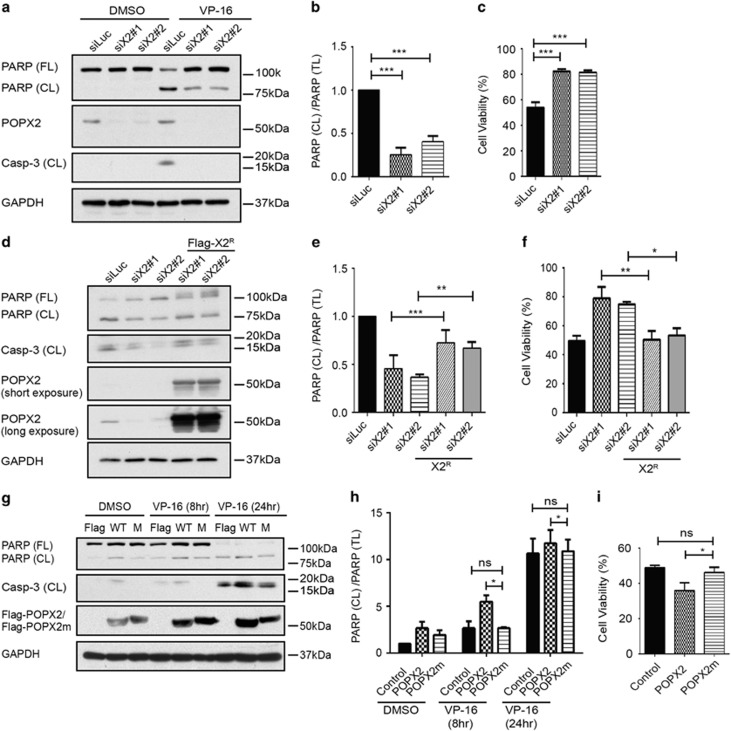
POPX2 regulates VP-16 induced apoptosis. (**a**) U-2OS cells were transfected with luciferase siRNA (siLuc) or POPX2 siRNAs (siX2#1 and siX2#2) as indicated for about 48 h, followed by treatment with DMSO or VP-16 (40 μg/ml) for another 24 h. Full-length PARP [PARP (FL)], cleaved PARP [PARP (CL)], cleaved capspase-3 [Casp-3 (CL)] and POPX2 were analyzed by immunoblotting. (**b**) Analysis of PARP (CL) versus total PARP [PARP (TL)] in cells transfected with siLuc or siX2 (siX2#1 and siX2#2) after VP-16 treatment. Intensities of PARP (CL) and PARP (FL) were measured from Western blots using ImageJ. Analysis was done by calculating the relative ratio of PARP (CL)/[PARP (CL) + PARP (FL)]. (**c**) Cell viability was measured by calculating the percentage of cells not stained by trypan blue. (**d**-**f**) U-2OS cells with POPX2-knockdown were transfected with POPX2 cDNA construct resistant to the siRNAs to restore POPX2 levels, followed by VP-16 treatment for 24 hours. Cleavage of PARP and Caspase-3, and expression of POPX2 were analyzed by Western blot (**d**). Relative ratio of PARP (CL) versus PARP (TL) (**e**), and cell viability 24 hours after VP-16 treatment (**f**) were calculated as mentioned in (**b**) and (**c**), respectively. (**g**-**i**) U-2OS cells transfected with the following constructs: Flag vector (control), wildtype POPX2 (WT) or the phosphatase-dead mutant of POPX2 (POPX2m or M) were treated with or without VP-16 for 8 or 24 hours. The cell lysates were then assayed to detect cleavage of PARP and Caspase-3, and levels of POPX2 and POPX2m by immunoblotting (**g**); the ratio of PARP (CL)/PARP (TL) (**h**) and cell viability (**i**) were calculated as in (**b**) and (**c**), respectively. The results in (**b**), (**c**), (**e**), (**f**), (**h**) and (**i**) are presented by mean ± S.E. (error bar), and represent at least three independent experiments. *p≤0.05, **p≤0.01, ***p≤0.001, as analyzed by Student’s *t*-test.

To delineate the functions of TAK1-POPX2 interaction, we investigated possible roles of POPX2 in cell death in TAK1 related context. As TAK1 has been reported to be essential for cell survival^32, 33^ and our preliminary data suggest that POPX2 can dephosphorylate and possibly downregulate TAK1 (Figure 2), we are curious to find out whether POPX2 affects TAK1’s anti-apoptotic function when the cell is challenged by genotoxic compounds. In the following experiments, we used etoposide (VP-16) which causes DNA double stranded breaks to induce apoptosis. VP-16 is a topoisomerase inhibitor and has been used as a cytotoxic anti-cancer drug. We transfected U-2OS cells with either luciferase siRNA or POPX2 siRNA. As indicated by the cleavage of caspase-3 and its substrate PARP, cells with higher levels of POPX2 were more vulnerable to VP-16 treatment (Figures 3a and b). Cells with POPX2-knockdown exhibited higher survival post VP-16 stimulation (Figure 3c). When we introduced siRNA-resistant POPX2 into the POPX2-knockdown cells, we found that restoration of POPX2 levels decreased the resistance to apoptosis (Figures 3d and f). These observations suggest that silencing POPX2 promotes survival for cells suffering DNA damage. As POPX2 is a Ser/Thr phosphatase, we next determined if the phosphatase activity of POPX2 was required for the regulation of apoptosis. We found that overexpression of wildtype POPX2 promoted apoptosis, whereas cells expressing the phosphatase-dead mutant of POPX2 did not show obvious increase in apoptosis (Figures 3g–i). Hence, POPX2 is able to regulate VP-16-induced apoptosis.

As silencing POPX2 shows decreased sensitivity to VP-16 induced cell death, we are curious to know if chemoresistant cancer cells show decreased levels of POPX2. The expression of POPX2 were examined in different pairs of chemoresistant cancer cells and their respective parental cells. Interestingly, higher levels of POPX2 were observed in multi-drug resistant cells such as HL-60/MX2 and TC7 than their parental cells HL-60 (human promyelocytic leukemia cells) and Caco-2 (human colorectal adenocarcinoma cells), respectively. Lower POPX2 levels were observed in H69AR cells (adriamycin-resistant) compared to the parental line NCI-H69 (human small cell lung tumor cells). Similarly, lower POPX2 levels were found in ovarian cancer cell line A2780cisR, which is resistant to cisplatin (Supplementary Figure S3). Our observations suggest that the development of chemoresistance is more complex than modulating protein levels.

### Activation of TAK1-IKK*β* axis in the POPX2 knockdown cells contributes to cell survival upon DNA damage

**Figure 4:**
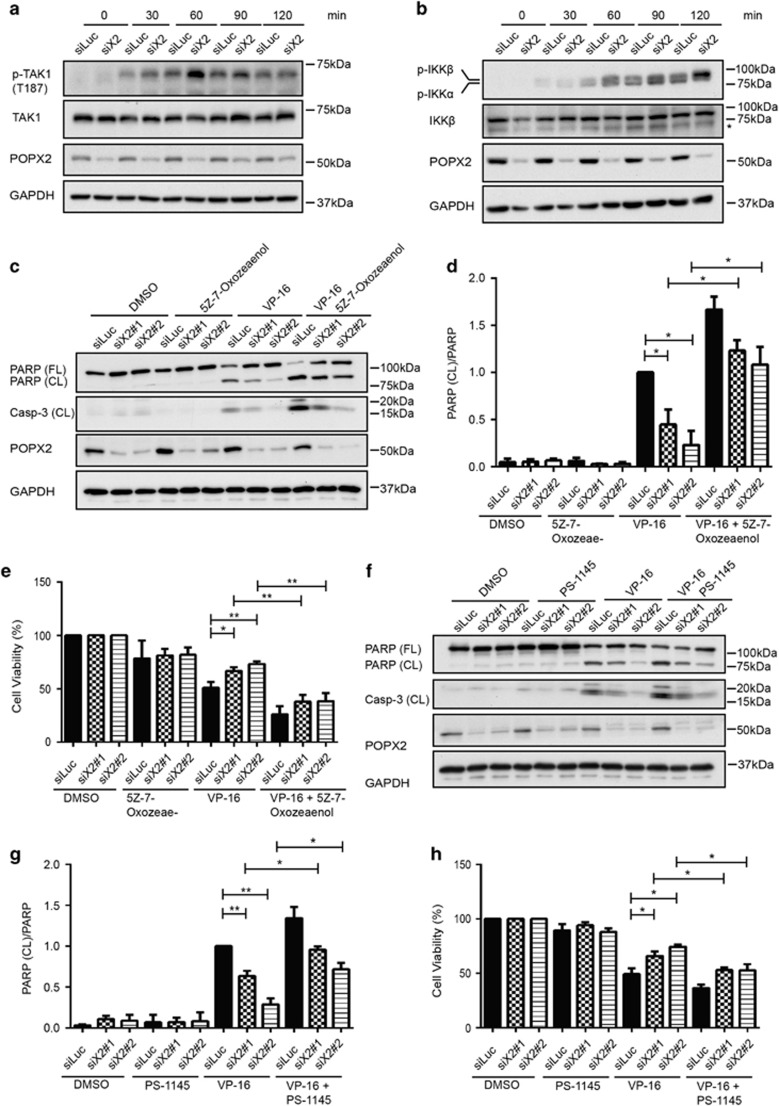
Activation of TAK1-IKK axis promotes survival of POPX2-knockdown cells. (**a**) U-2OS cells were transfected with siLuc or siX2#2 for about 48 h, followed by stimulation of VP-16 at indicated time points. Phophorylation of TAK1 at Thr187, levels of TAK1 and POPX2 were examined by Western blot. (**b**) Cells were treated as in **a**. Phosphorylation of IKK*α* and IKK*β* at Ser176 and Ser177 respectively, levels of IKK*β* and POPX2 were detected by immunoblotting. * indicates non-specific band detected by anti-IKK*β* antibody. (**c**–**e**) Cells transfected with siLuc, siX2#1, and siX2#2 were pre-treated with or without TAK1 inhibitor 5Z-7-Oxozeaenol (1 *μ*M) for 3 h, followed by treatment of VP-16 or DMSO for another 24 h in presence of the inhibitor. Cleavage of PARP, caspase-3, and protein levels of POPX2 are detected by immunoblotting (**c**). Relative ratios of PARP (CL)/PARP (TL) (**d**) were calculated as stated in Figure 3b. The viability (**e**) of the cells were measured using MTT assays. Cell viability was presented as relative ratios of MTT absorbance of the treated cells compared with the respective untreated cells. (**f**–**h**) Cells transfected with siLuc, siX2#1, and siX2#2 were pre-treated with or without IKK inhibitor PS-1145 (30 *μ*M) for 3 h, followed by treatment of VP-16 or DMSO for another 24 h in presence of the inhibitor. Cleavage of PARP, caspase-3, and levels of POPX2 were determined by Western blot (**f**). Relative ratios of PARP(CL)/PARP(TL) (**g**) and cell viability (**h**) were measured as mentioned in (**d**,**e**). The results in (**d**, **e**, **g** and **h**) are presented as mean±S.E. (error bar), and represent at least three independent experiments. *P⩽0.05, **P⩽0.01, as analyzed by Student’s *t*-test

Genotoxic stress is able to stimulate fast and transient activation of TAK1, which triggers a series of self-defense mechanisms to protect the cell from apoptosis.^34, 35, 36^ Since POPX2 affects the phosphorylation and hence the activation of TAK1, we wondered if the effects of POPX2 on VP-16 induced apoptosis are dependent on TAK1 signaling. As expected, DNA damage resulted from VP-16 treatment stimulated rapid changes in TAK1 activation, which was indicated by increased phospho-TAK1 (Thr187) levels that peaked at 60 min after VP-16 treatment, followed by gradual reduction in phospho-TAK1 levels (Figure 4a). We also observed higher phospho-TAK1 levels in POPX2 knockdown cells than in the control cells (Figure 4a). This may suggest higher TAK1 activities in cells with lower levels of POPX2. TAK1 directly phosphorylates IKK*β*,^22, 23^ hence phosphorylation of IKK*β* can also serve to indicate activation of its upstream kinase TAK1. To provide further support that higher TAK1 activity was observed in cells with POPX2-knockdown in response to DNA damage, we therefore checked phospho-IKK levels upon VP-16 stimulation. Consistent with our earlier observations with phospho-TAK1, higher phospho-IKK*β* levels were also observed in the POPX2 knockdown cells (Figure 4b). However, phospho-IKK*β* levels kept increasing till 120 min post VP-16 stimulation, suggesting slower attenuation of IKK*β* signaling compared to that of TAK1. Taken together, silencing POPX2 facilitates phosphorylation of IKK*β* by TAK1 in response to VP-16.

Our initial observations suggest that POPX2 knockdown cells show less apoptosis and higher TAK1 activity when treated with VP-16. We thus investigated possible roles of TAK1 in this context. We blocked activation of TAK1 using the inhibitor 5Z-7-Oxozeaenol (Supplementary Figure S4a). As expected, the POPX2 knockdown cells with inhibition of TAK1 activity exhibited more cleavage of PARP as well as caspase-3 than their respective counterparts (Figures 4c and d). Inhibition of TAK1 also reduced the survival of POPX2 knockdown cells (Figure 4e). Similar results were obtained when the POPX2 knockdown cells were treated with IKK inhibitor PS-1145 to block the activation of IKK upon VP-16 stimulation (Figures 4f–h and Supplementary Figure S4b). Thus, the TAK1-IKK axis suppresses VP-16 induced apoptosis of the POPX2 knockdown cells, suggesting possible roles of POPX2 in the regulation of TAK1-IKK pathway.

### POPX2 regulates NF-*κ*B-mediated gene transcription in response to DNA damage

**Figure 5:**
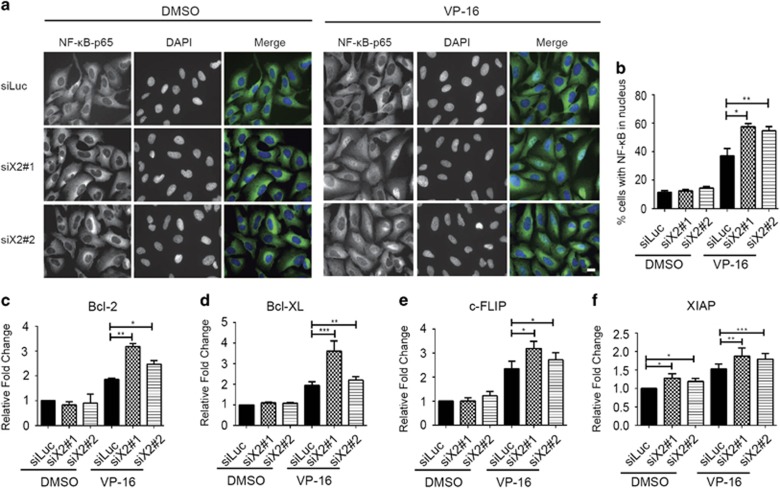
POPX2 knockdown promotes NF-*κ*B-mediated gene transcription. (**a**) U-2OS cells were transfected with siLuc, siX2#1 and siX2#2 for 48 h, followed by stimulation with DMSO or VP-16 for 4 h. The cells were fixed and stained with NF-*κ*B-p65 antibody and DAPI to visualize NF-*κ*B and the nucleus, respectively. Scale bar: 20 *μ*m. (**b**) Percentage of cells with NF-*κ*B in the nucleus was calculated by dividing the number of cells containing nuclear NF-*κ*B with total number of cells counted. Quantified data were presented as mean±S.E. (error bar) from three independent experiments (*N*=100). *P⩽0.05, **P⩽0.01, as analyzed by Student’s *t*-test. (**c**–**f**) U-2OS cells were first transfected with respective siRNAs as indicated for 48 h, and subsequently treated with VP-16 for 24 h. Transcript levels of Bcl-2 (**c**), Bcl-XL (**d**), c-FIP (**e**) and XIAP (**f**) in cells treated with or without VP-16 were compared using real-time PCR. Quantified data are displayed as mean±S.E. (error bar) from three individual experiments. *P⩽0.05, **P⩽0.01, ***P⩽0.001, as analyzed by Student’s *t*-test

Translocation of NF-*κ*B from the cytosol to the nucleus depends on the phosphorylation status of I*κ*B that sequesters NF-*κ*B in the cytosol. Proteasome-mediated degradation of I*κ*B due to its phosphorylation by the upstream IKK kinase leads to the release and activation of NF-*κ*B, which is indicated by the translocation of NF-*κ*B into the nucleus.^24^ Since the activation of TAK1-IKK axis contributes to reduced apoptosis in POPX2 knockdown cells, we determined the effects of POPX2 knockdown on the activation of NF-*κ*B. Localization of NF-*κ*B in response to DMSO and VP-16 treatment was determined by immuno-staining of p65, a component of the NF-*κ*B complex. There is no obvious difference in the distribution of NF-*κ*B in both control and POPX2-knockdown cells treated with DMSO (control). However, POPX2-knockdown cells showed more NF-*κ*B nuclear localization compared to control cells after VP-16 treatment (Figures 5a and b). Thus, silencing POPX2 promotes the translocation of NF-*κ*B from the cytosol to the nucleus in response to DNA damage. As the localization of NF-*κ*B in the nucleus indicates increased transcription of targeted genes, we then proceeded to determine whether POPX2 can affect the transcript levels of anti-apoptotic genes regulated by NF-*κ*B. We thus determined the levels of a few reported NF-*κ*B regulated genes such as Bcl-2, Bcl-XL, c-FLIP and XIAP.^37, 38, 39, 40^ No observable differences in the mRNA levels of Bcl-2, Bcl-XL and c-FLIP were seen in DMSO treated POPX2-knockdown and control cells. We found that the mRNA levels of XIAP in POPX2 knockdown cells were slightly higher than those in control cells without stimulation. VP-16 treatment promoted the transcription of all four genes tested with POPX2-knockdown cells exhibiting significantly higher mRNA levels of these anti-apoptotic genes after 24 h of drug treatment (Figures 5c–f). These results demonstrated that POPX2 promotes apoptosis by affecting NF-*κ*B-mediated gene transcription in response to VP-16.

Consistent with the obervations for mRNA, cells with POPX2 knocked down showed higher Bcl-XL XIAP, c-FLIP and Bcl-2 protein levels than those in the control cells 24 h after VP-16 treatment (Supplementary Figure S5a and b). Interestingly, the overall levels of these anti-apoptotic proteins were found to be lower significantly in the control cells after VP-16 treatment (Supplementary Figure S5a and b). To rule out the possibility that it was due to increased POPX2 activity after VP-16 treatment, which might contribute to the decreased levels of these anti-apoptotic proteins in the cells, *in vitro* phosphatase assay was performed with POPX2 purified from cells treated with either DMSO or VP-16. No obvious difference in the phosphatase activity was observed (Supplementary Figure S5c), suggesting that there might be post-translational regulation that contributed to the decreased levels of these anti-apoptotic proteins in reponse to VP-16 treatment.

## Discussion

The balance of anti- *versus* pro-apoptotic signaling determines cell survival when the cells are under stress. In this study, we have uncovered a role for POPX2 as a negative regulator of TAK1 signaling. We also demonstrated that reducing POPX2 levels enhances resistance to DNA-damage induced apoptosis by upregulating anti-apoptotic factors.

The activation of TAK1 is rapid and transient in response to inflammatory stimulation as well as other cellular stresses. While TAK1 is activated within 20 min when induced by IL-1 and LPS, triggering TAK1 activity by TNF-*α* requires less than 5 min.^41, 42^ However, the kinase activity of TAK1 returns to basal levels within a few hours after activation. We also observed rapid activation of TAK1 when the cells are treated with VP-16. The kinase activity of TAK1 gradually reduces after 60 min as indicated by decreased phospho-TAK1 levels (Figure 4a). The rapid upregulation and then downregulation of TAK1 activity suggests possible molecular mechanisms that prevent prolonged activation of TAK1. Indeed, several protein phosphatases including PP2A, PP1, PP6, PP2C*β* and PP2C*ε* have been identified as negative regulators of TAK1.^27, 30, 31, 43, 44^

Here, we report another PP2C family protein phosphatase POPX2 that negatively regulates TAK1 by binding to TAK1 and dephosphorylates TAK1-Thr187. The phosphorylation sites required for TAK1 activation which are found in the activation loop include Thr178, Thr184, Thr187 and Ser192; among which Thr187 is recognized as the most important phosphorylation site.^29, 45^ It is not clear if all the sites are phosphorylated during TAK1 activation. In this study, we observed higher phosphorylation levels of TAK1 and its substrates IKK*β* upon treatment with VP-16 in POPX2 knockdown cells (Figures 4a and b). We have also demonstrated that POPX2 can directly dephosphorylate TAK1 (Figures 2b and c). It is possible that POPX2 functions to inhibit TAK1 activity in the cell and silencing POPX2 allows increased TAK1 activation. It is well-known that TAK1 regulates NF-*κ*B-mediated transcription via IKK complexes.^34, 35^ In POPX2 knockdown cells, elevated nuclear translocation of NF-*κ*B and increased mRNA levels of NF-*κ*B-mediated genes (Figures 5a–f) further support our hypothesis that POPX2 negatively regulates TAK1-IKK-NF-*κ*B signaling.

Interestingly, although we observed dramatic increase of the anti-apoptotic genes at transcript levels after VP-16 treatment in both control and POPX2-knockdown cells (Figures 5c–f), there was little elevation of the respective protein levels after VP-16 treantment. Nevertheless, POPX2-knockdown cells showed higher levels of the anti-apoptotic proteins compared to the control after VP-16 treatment, which is consistent with higher mRNA levels of anti-apoptotic genes observed in POPX2-knockdown cells (Figure 5 and Supplementary Figure S5). Our observations suggest that in addition to transcriptional modulation, there might also be post-translational regulation in response to apoptosis.

**Figure 6:**
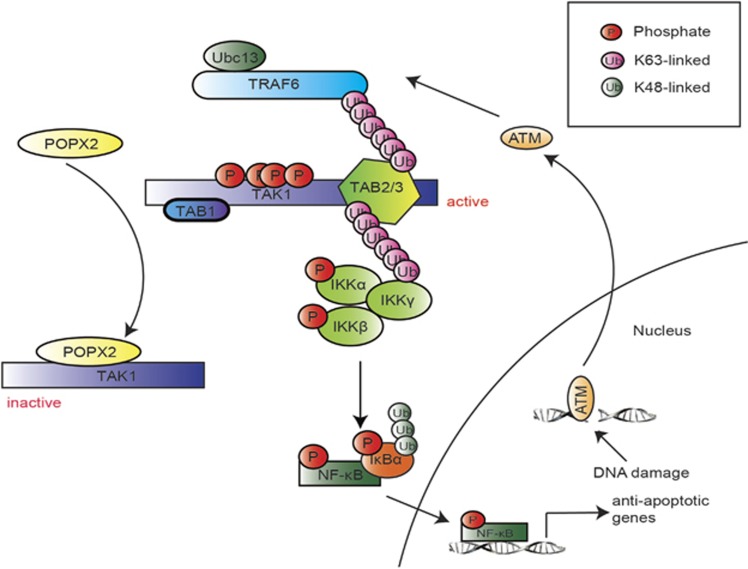
Proposed working model: POPX2 regulates the TAK1-IKK-NF-*κ*B pathway during DNA-damage-induced apoptosis. DNA damage stimulates the activation and translocation of ATM to the cytosol, which promotes the formation of a complex containing TRAF, TAK1 and its binding proteins. TAK1 is activated as a consequence. Activated TAK1 then activates downstream signaling pathway through the IKK complex resulting in upregulation of NF-*κ*B-mediated transcription for anti-apoptotic genes. POPX2, by acting directly on TAK1, impedes the activation of TAK1. As a result, downstream anti-apoptotic activities in response to DNA damage are inhibited

Two known substrates of POPX2, PAK and CaMKII, are reported to promote cell survival,^46, 47, 48, 49^ thus implicating POPX2 in the regulation of cell survival and apoptosis. Indeed, overexpression of POPX2 in mammalian cells enhances apoptosis.^50^ Nevertheless, the exact mechanism of POPX2 in the regulation of apoptosis remains unknown. In this study, we found that POPX2 negatively regulates TAK1 signaling, which is why POPX2-knockdown cells are more resistant to apoptosis (Figure 6). TAK1 is an important pro-survival regulator. Cells lacking TAK1 are vulnerable to cell death induced by VP-16, ionizing radiation and TNF-*α*.^32, 36, 51^ When the cells suffer DNA damage, a series of self-defense programs are activated to rescue the cells from apoptosis. Ataxia telanglectasia mutated (ATM) kinase is activated by the MRN (Mre1, Rad50 and Nbs1) complex, which senses double stranded DNA breaks.^52, 53^ ATM translocates rapidly to the cytosol to activate TRAF via a TRAF-binding motif.^34^ This then promotes the activation of TAK1 and its subsequent downstream kinase IKK*β*. Activated IKK*β*, in association with IKK*α* and IKK*γ*, initiates the release and translocation of NF-*κ*B to the nucleus, which in turn, upregulates anti-apoptotic genes to protect cells from apoptosis. Association of POPX2 with TAK1 hampers the activation of the kinase as POPX2 dephosphorylates residues essential for TAK1 activation. Silencing POPX2, on the other hand, promotes TAK1 signaling as this negative regulation is removed. Thus, more active TAK1 contributes to higher levels of anti-apoptotic proteins through the IKK-NF-*κ*B pathway and tilts the balance in favor of cell survival. Consequently, POPX2-knockdown cells are more resistant to VP-16-induced apoptosis.

Resistance to chemotherapy contributes to treatment failure and cancer relapse. There are many factors that lead to chemoresistance, one of them being the selection of cancer cells with increased pro-survival signaling. Increased ERK and p38 MAPK signaling have been reported to contribute towards chemoresistance.^54, 55^ There are also reports which suggest that inhibition of GSK3 might help the cells to overcome chemoresistance.^56, 57^ Our earlier studies showed that invasive cancer cells have higher levels of POPX2 and that cells with high levels of POPX2 also exhibit higher ERK activities.^6, 8^ We also found that knocking down POPX2 leads to lower GSK3 activities. These earlier observations may suggest that POPX2 could be a possible target for therapeutic intervention. However, our current work shows that POPX2 can downregulate TAK1 and affect the anti-apoptotic activities of TAK1, implying that silencing POPX2 could facilitate TAK1 activation and will lead to increased cell survival. It appears that downregulation of POPX2 may be effective in blocking cell motility and invasiveness at the initial stages of metastasis where cell migration is required. However, lower levels of POPX2 may enhance cancer cell’s chemoresistance and favor tumor growth due to the upregulation of TAK1 activities. Indeed, patients with lower levels of POPX2 showed lower overall survival rate and lower relapse-free survival.^9^ Therefore, a combination therapy targeting TAK1-IKK-NF-*κ*B pathway may help to increase the effectiveness of cancer treatment.

## Material and methods

### Reagents

Rabbit polyclonal antibodies against Bcl-2, Bcl-XL, cleaved Caspase-3, His, IKK*β*, PARP and rabbit monoclonal antibodies against phospho-IKK*α*/IKK*β* (S176/177), p65, TAB1, phospho-TAK1 (T187), and TAK1 were purchased from Cell Signaling Technology (Danvers, MA, USA). Mouse monoclonal antibodies against actin and GAPDH were from Millipore and Thermo Fisher Scientific (Waltham, MA, USA), respectively. Mouse monoclonal antibody and rabbit polyclonal antibody against Flag were purchased from Sigma-Aldrich (St. Louis, MO, USA). Mouse monoclonal antibody against XIAP was from BD Biosciences (Franklin Lakes, NJ, USA). Rabbit polyclonal antibody against phospho-Ser/Thr was from ECM Biosciences (Versailles, KY, USA). Rabbit polyclonal antibodies against GFP and GST were from Thermo Fisher Scientific and Bethyl Laboratories Inc. (Montgomery, TX, USA), respectively. Rabbit polyclonal antibody against c-FLIP, Mouse polyclonal antibody against POPX2 were from Abcam (Cambridge, UK), and rabbit anti-sera against POPX2 were from Koh Lab. TrueBlot Ultra anti-mouse Ig HRP and TrueBlot anti-rabbit IgG HRP were obtained from Rockland (Limerick, PA, USA). Constructs of POPX1, POPX2 and POPX2m were generated as described previously.^1^ GST-tagged full length of PP2C*β* (NM_002706.5) was generated by PCR from human cDNA and cloned in pXJ vector. Full length of TAK1 (NM_003188.3) and TAB1 (NM_006116.2) were generated by cloning and inserted into either pXJ vector for protein expression in mammalian cells or pGEX-6P-1 vector for protein expression in bacteria. Deletion mutant of GST-tagged TAK1 were constructed by PCR with different pairs of primers and cloned into pXJ vector. The sequences of all the Stealth RNAi siRNAs (Thermo Fisher Scientific) used in this study are listed in Supplementary Table 1.

### Cell culture and transfection

HEK293, COS-7 and U-2OS cells were cultured in Dubecco’s modified Eagle’s medium (DMEM), high glucose (Sigma-Aldrich) supplemented with 3.7 g/l sodium bicarbonate and 10% FBS. Caco-2 and TC-7 cells were grown in DMEM, high glucose supplemented with 3.7 g/l sodium bicarbonate, 1% non-essential amino acids (NEAA) and 20% FBS. A2780, HL-60 and HL-60/MX2 cells were maintained in medium RPMI 1640 (Sigma-Aldrich) supplemented with 2 g/l sodium bicarbonate and 10% FBS. A2780cisR cells was cultured in RPMI 1640 medium supplemented with 2 g/l sodium bicarbonate, 10% FBS and 1 *μ*M cisplatin. All mammalian cells were grown in a humidified incubator with 5% CO_2_ at 37 °C. Transfection was carried out using Lipofectamine 2000 according to the manufacture’s protocol (Thermo Fisher Scientific).

### SDS-PAGE and western blot

Proteins were resolved on 8–12% SDS-PAGE. After separation, the proteins were transferred to nitrocellulose membrane (Bio-Rad, Hercules, CA, USA). For protein phosphorylation analysis, Mn^2+^-based phos-tag SDS-PAGE gel containing 20 μM of phos-tag (Wako, Osaka, Japan) and 0.1 mM MnCl_2_ was used according to the manufacturer’s protocol. The transferred membrane was incubated with primary antibody overnight at 4 °C, and then incubated with HRP-conjugated secondary antibody for 1 h at room temperature. The chemiluminescent signal of each blot was detected using Novex ECL substrate (Thermo Fisher Scientific) or Lumigen ECL Ultra (GE).

### Immunoprecipitation

The cells were lysed in protein extraction buffer containing 20 mM Tris, pH 7.4, 150 mM NaCl, 0.5% Triton X-100 and protease inhibitor. The lysates were homogenized by passing through 29G syringe several times. After centrifugation, the cleared lysates were incubated with Glutathione Sepharose 4B^TM^ (GE) beads, ANTI-FLAG M2 Affinity Gel (Sigma-Aldrich), or indicated antibodies for 2 to 12 h at 4 °C. The complexes recovered were washed three times with buffer containing 20 mM Tris, pH 7.4, 250 mM NaCl, and 0.5% Triton X-100, and analyzed by western blot.

### Protein binding assay

Proteins used in the binding assay were all expressed in Rosetta competent cells. His-POPX2 or His-POPX2m was incubated with GST, GST-TAK1, or GST-TAB1 bound to glutathione beads, followed by stringent wash (three times) with buffer containing 25 mM Tris, pH 7.4, 300 mM NaCl and 1% Triton X-100, 1 mM EDTA. The bound complexes were eluted in 1 × SDS sample buffer and subjected to western blot analysis.

### *In vitro* phosphatase assay

Phospho-Flag-TAK1 was purified from HEK293 cells co-expressing Flag-TAK1 and HA-TAB1, whereas Flag-TAK1 was purified from HEK293 cells transfected only with construct containing Flag-TAK1. For the phosphatase assay, phosphorylated TAK1 was incubated with various phosphatases in phosphatase buffer containing 20 mM Tris, pH 7.5, 10 mM MgCl_2_, 5 mM MnCl_2_, 1 mM DTT and protease inhibitor for 45 min at 30 °C. The reactions were terminated by addition of SDS sample buffer.

### MTT assay

About 3000 cells were seeded in standard 96-well plates in 100 *μ*l of complete DMEM for 24 h. After respective drug treatment, the culture medium was replaced with 100 *μ*l complete DMEM containg 0.5 mg/ml 3-(4,5-dimethylthiazol-2-yl)-2,5-diphenyl tetrazolium bromide (MTT) and the plates were incubated at 37 °C for 2 h. The medium was aspirated, and MTT crystals were solubilized in 200 *μ*l of DMSO. The OD was measured on TECAN Infinite 200 Pro microplate reader using 570 nm as test wavelength and 630 nm as reference wavelength.

### Immunofluorescence staining

Cells were fixed with 4% paraformaldehyde, permeabilized in 0.2% Triton X-100 and blocked with 4% BSA. The cells were then incubated with primary antibody in 1% Triton X-100 at 4 °C overnight, followed by incubation with secondary antibody in 1% Triton X-100 for 1 h at room temperature. Coverslips were mounted using Vectorshield with DAPI (Vector Laboratories, Burlingame, CA, USA) and visualized on AxioObserver Z1 Microscope (Zeiss, Oberkochen, Germany) with Plan-Apochromat × 40 1.25 objective. Images were captured using a Roper Scientific CoolSNAP CCD camera.

### Real-time quantitative PCR analysis

Total RNA was extracted using RNeasy mini kit (Qiagen, Hilden, Germany), followed by synthesis of cDNA using SuperScript VILO cDNA synthesis kit (Thermo Fisher Scientific), as per the manufacturer’s protocol. Real-time PCR was performed using StepOnePlus real-time PCR system (Thermo Fisher Scientific) with SYBR Green real-time PCR master mixes (Thermo Fisher Scientific) according to the manufacturer’s instruction. The relative gene expression level was calculated using 2^−ΔΔCt^ Method. Expression of the target gene was normalized against GAPDH values, and presented in relative to their corresponding controls. The primers used in this study are listed in Supplementary Table 1.
