# Supplementary material for: POPX2 phosphatase regulates apoptosis through the TAK1-IKK-NF-κB pathway
Source: Cell Death Dis. 2017 Sep 14;8(9):e3051–. doi: 10.1038/cddis.2017.443 (PMC5636987; doi:10.1038/cddis.2017.443)
Supplement: Supplementary Materials [file cddis2017443x1.pdf]

Supplementary Materials
One table and 5 figures

Table 1

The sequences of Stealth RNAi siRNAs used in this study are listed below:

Luciferase siRNA (siLuc)	sense: 5'-ACAUCACGUACGCGGAAUACUUCGA-3'
	antisense: 5'-UCGAAGUAUUCCGCGUACGUGAUGU-3'
POPX2 siRNA#1 (siX2#1)	sense: 5'-ACCGCGCCUACUUUGCUGUGUUUGA-3'
	antisense: 5'-UCAAACACAGCAAAGUAGGCGCGGU-3'
POPX2 siRNA#2 (siX2#2)	sense: 5'-CCAAGAUGACCUGUUGUGUCAUAUA-3'
	anti-sense: 5'-UAUAUGACACAACAGGUCAUCUUGG-3'
POPX2 siRNA#1 targets the coding sequence of POPX2, siRNA#2 targets the 3' UTR region of POPX2 mRNA	

The sequences of primers used in the real-time PCR quantitative assay are listed below:

Bcl-XL	For: 5'- GAGCTGGTGGTTGACTTTCTC -3'
	Rev: 5'- TCCATCTCCGATTCAGTCCCT -3'
Bcl-2	For: 5'- GGTGGGGTCATGTGTGTGG-3'
	Rev: 5'- CGGTTCAGGTACTCAGTCATCC-3'
c-FLIP	For: 5'- ATTTGCCTGTATGCCCGAGC -3'
	Rev: 5'- CCTGAGTGAGTCTGATCCACAC -3'
XIAP	For: 5'- TATCAGACACCATATACCCGAGG -3'
	Rev: 5'- TGGGGTTAGGTGAGCATAGTC -3'
GAPDH	For: 5'-GTGGTCTCCTCTGACTTCAACAG -3'
	Rev: 5'-CTGTAGCCAAATTCGTTGTCATAC -3'

Figure S1

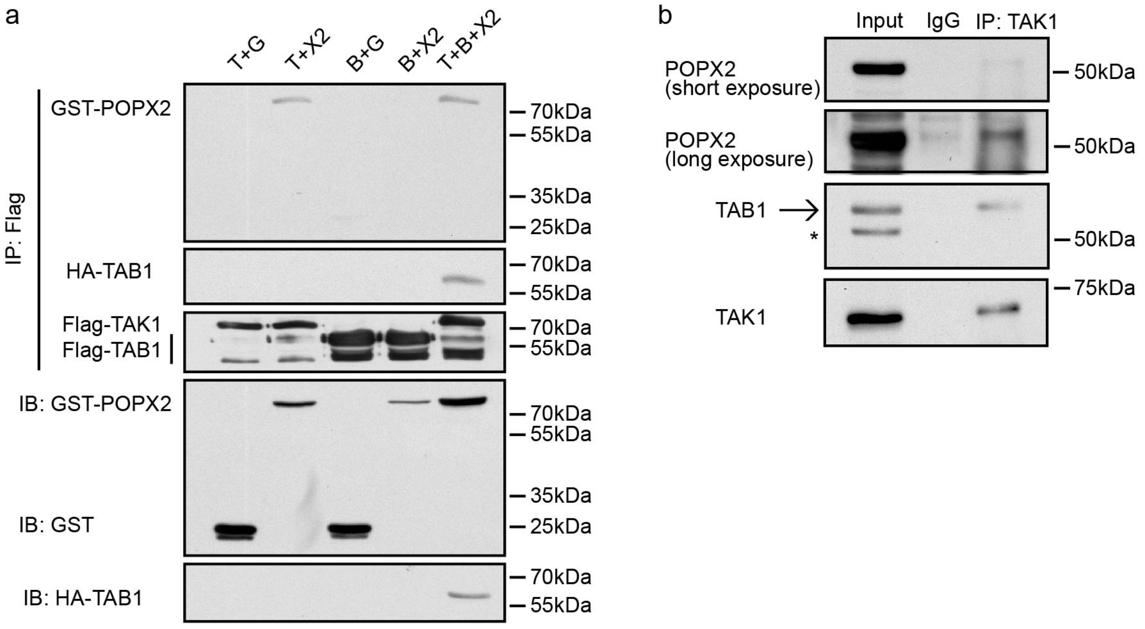

Figure S1. Association of POPX2 with TAK1. (a) POPX2 is found in the same complex as TAK1. GST or GST-POPX2 was co-expressed with Flag-TAK1, Flag-TAB1 or Flag-TAK1 and HA-TAB1 in COS-7 cells. Proteins immunoprecipitated by Flag beads were subjected to Western blot analysis. (b) Endogenous interaction of POPX2 with TAK1 and TAB1. Endogenous TAK1 was recovered with anti-TAK1 sheep antibody from U-2OS cells. The co-immunoprecipitated POPX2 and TAB1 were detected with their respective antibodies. * indicates POPX2.

Figure S2

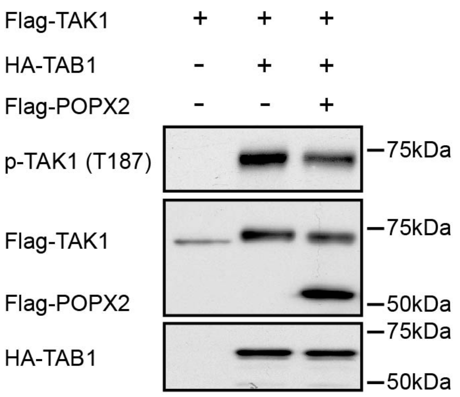

Figure S2. POPX2 dephosphorylates TAK1 at Threonine 187. Flag-TAK1 was expressed alone, co-expressed with HA-TAB1, or with HA-TAB1 and Flag-POPX2. The cells were harvested 24 hours after transfection, and the cell lysates were subjected to Western blot analysis.

Figure S3

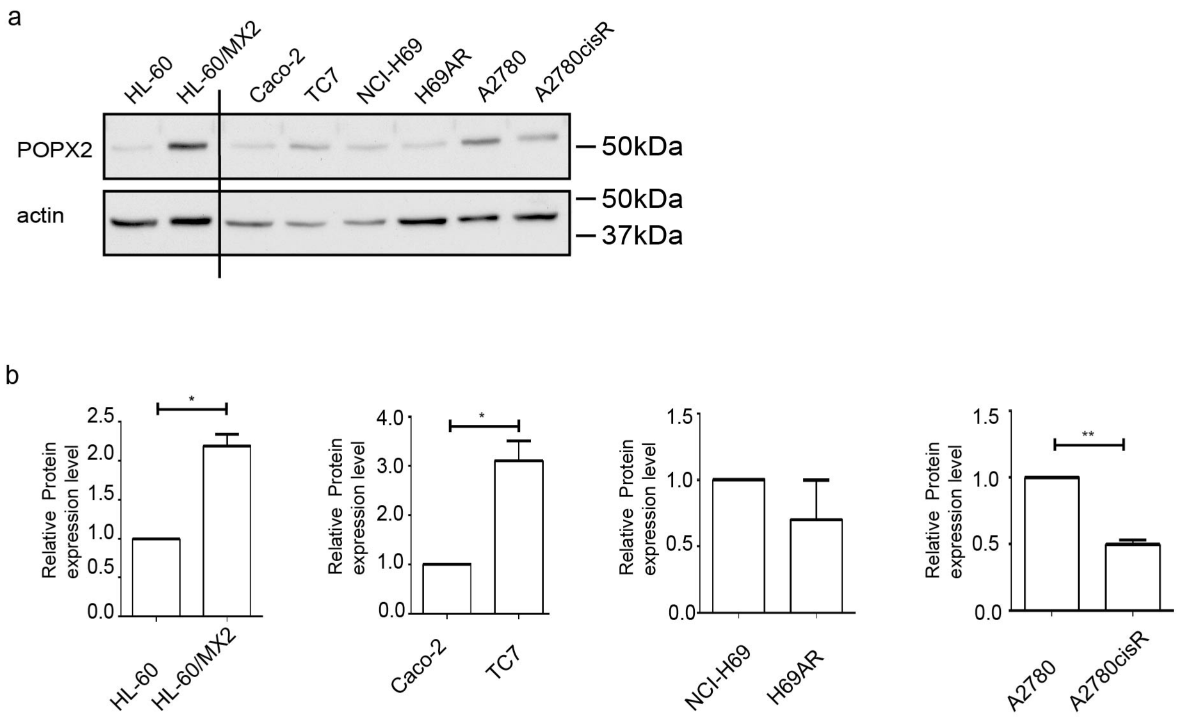

Figure S3. (a) Western blot analysis of POPX2 levels in different chemoresistant cells and the respective parental cells. Total cell lysates were harvested from different cancer cell lines. Equal amounts of proteins were used for SDS-PAGE and Western blot analysis with anti-POPX2 antibody and anti-actin antibody. (b) Densitometry analysis of protein levels of POPX2 in different chemoresistant cells and the respective parental cells. The levels of POPX2 were normalized against the levels of actin. The results in (b) are presented as mean ± S.E. (error bar), and represent at least three independent experiments. * $p \leq 0.05$, ** $p \leq 0.01$, as analyzed by students' t-test.

Figure S4

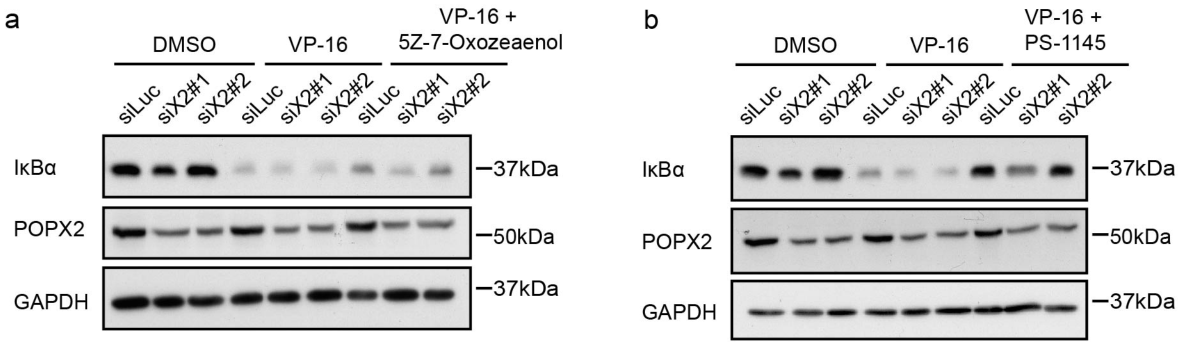

Figure S4. (a) Inhibition of TAK1 blocks IκBα degradation caused by VP-16 treatment. U-2OS cells were pre-treated with 1μM TAK1 inhibitor 5Z-7-Oxozeaenol for 3 hours, followed by stimulation of VP-16 in the presence of the inhibitor for another 1 hour. Levels of IκBα were detected by immunoblotting. (b) Inhibition of IKK blocks IκBα degradation caused by VP-16 treatment. U-2OS cells were pre-treated with 30μM IKK inhibitor PS-1145 for 3 hours, followed by stimulation of VP-16 in the presence of the IKK inhibitor for another 1 hour. Protein levels of IκBα were determined by immunoblotting.

Figure S5

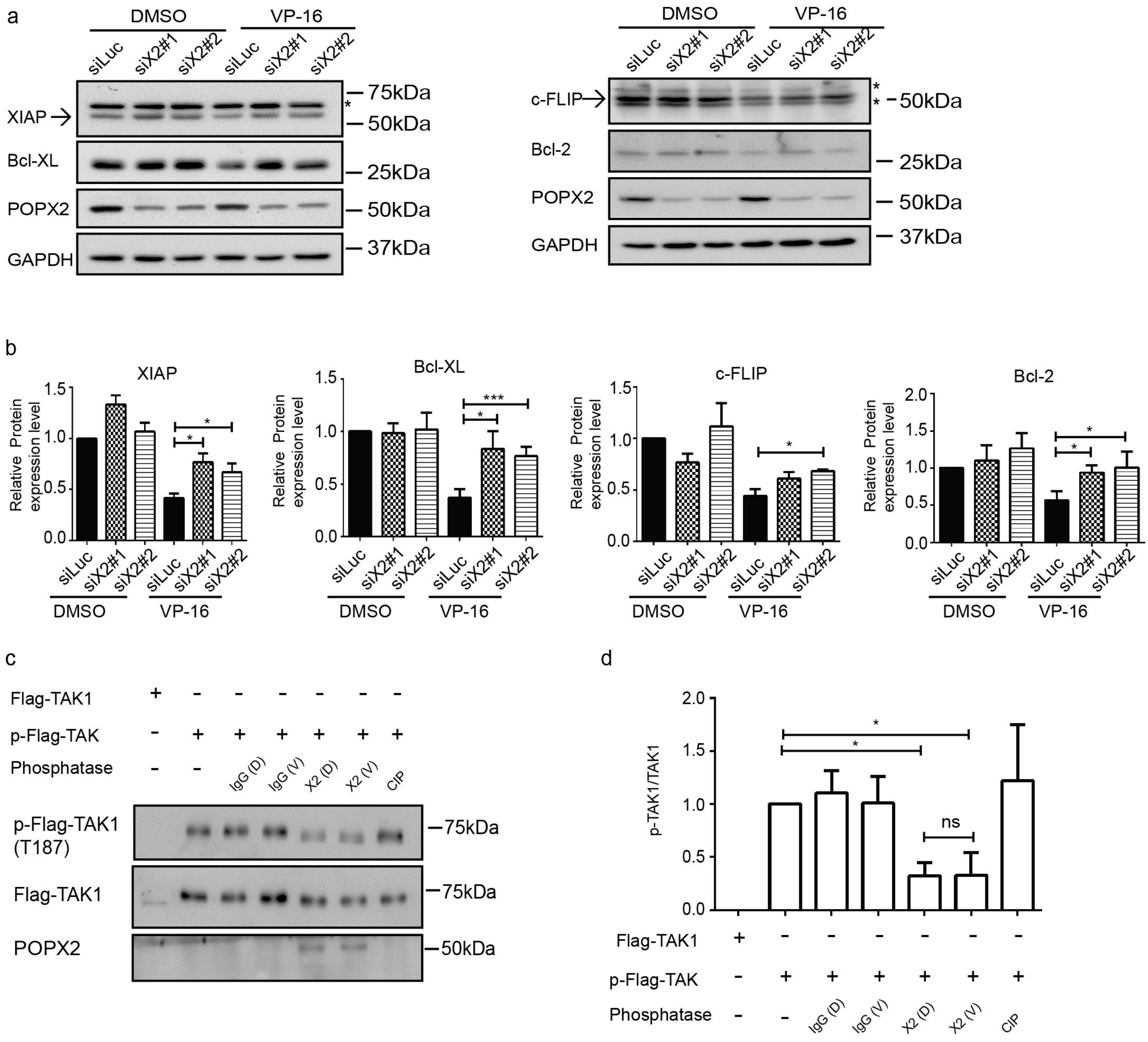

Figure S5. (a) Protein levels of XIAP, Bcl-XL, c-FLIP and Bcl-2 in cells treated with or without VP-16 for 24 hours. * indicates the non-specific bands detected. (b) Densitometry analysis of XIAP, Bcl-XL, c-FLIP and Bcl-2 protein levels normalized against GAPDH. (c) Flag-TAK1 or p-Flag-TAK1 was incubated with proteins purified from U-2OS cells with either mouse IgG or anti-PPM1F antibody in phosphatase buffer. The cells were treated with DMSO (D) or VP-16 (V) for 24 hours before they were assayed. Anti-p-TAK1 (T187) antibody was used to detect phosphorylation at threonine-187. Anti-Flag antibody was used to detect TAK1. Anti-POPX2 antibody was used to detect POPX2. TrueBlot® secondary antibody was used to avoid detection of IgG. (d) Bar chart showing relative ratio of p-TAK1 versus TAK1 from phosphatase assay. The results in (b) and (d) are presented as mean ± S.E. (error bar), and represent at least three independent experiments. * $p \leq 0.05$, ** $p \leq 0.01$, *** $p \leq 0.001$, ns (not significant) $p > 0.05$, as analyzed by students' t-test.
